# Health Tract, No. 227

**Published:** 1864-12

**Authors:** 


HEALTH TRACT, No. 227.

### THE FAITHLESS PHYSICIAN.

On Saturday the eighth day of October, 1864, a rich man was taken to his grave from the city of New York, where, by a life of industry, temperance, economy, and the exercise of a sound judgment, he had acquired a large estate; not suddenly, but in the progress of thirty years. From some obscure cause, he became a great sufferer from general neuralgia. A physician was called, but in the progress of weeks, did not afford the invalid that speedy and decided relief, which he and his friends so much desired. He wanted more potential remedies, and was not willing to submit to those restraints to system and rule in living, which the experience of years had taught his medical attendant were of the highest value ultimately. Another physician was called in, who advised a different course of procedure. He said the man needed strength and flesh and vigor; that these could only be secured by a generous diet and a liberal allowance of stimulants at every meal. Roast beef and the best brandy were regularly provided: the neuralgia was steadily abating and a speedy restoration was looked for. Still, days passed on, and gentlemen calling at his counting house were informed that, The Head of the firm was "out of town." This answered very well for a while, but a gentleman who had large business engagements with the invalid, found it absolutely necessary to save a ruinous loss, to have a personal interview; after a variety of obstacles were overcome the rich man was found at his own home in the city, the victim of the most fearful form of Delirium Tremens. Brandy had given relief; but it was soon found that unless it was administered more frequently, and in larger quantities the pains would return, hence there was an unbridled indulgence in the remedy, the result being, that at the end of a few weeks, it was found necessary to keep the patient always under the influence of liquor, that is, always drunk; at length however, the outraged stomach refused to retain the fiery stimulant a single moment, while for want of it a raving delirium sat in, ending in unconsciousness and death, before the business could be consummated by his intelligent signature. Here was a man who, temperate all his life, had died a drunkard's death as the result of an unwise medical prescription, the fashionable and almost universal prescription of a certain class of so called "Doctors," of "Bourbon Whiskey;" and there are thousands of deluded persons of every age and sex, especially in New England, on the same road to ruin. A few years ago, cod liver oil was given for everything; now it is Brandy. Shame on the medical colleges of the land, which send out annually so many incompetent and unprincipled graduates.

The American Tract Society, 150 Nassau Street, New-York, has published a 12mo. of 300 pages on "Christian Home Life," a book of examples and principles which is full of rich instruction for every parent and child in our country; also "Walter Martin, or the Factory, the School and the Camp;" "Madeline," by Rose Elmwood, is a sweet little book about farm life, a new home, a financial crisis, the drunkard's family, &c.; "The Bloom of Youth; or Worthy Examples," selected by the late Rev. Joseph Belcher; "Little Lucy of the West;" "A Little More;" "The Color Bearer;" "Rules for Holy Living, with questions for self examination; "Sixty Years in Sin," by Rev. Chas. J. Jones; "Something for the Locker," by Rev. Dr. J. B. Waterbury; "Remember," a word for soldiers, by Rev. A. W. Henderson, Chaplain of the 13th Illinois Cavalry, are all well worthy of being purchased as Christmas presents to children and our brave and self-denying soldiers in the field. But the most enduring and pleasure giving book of them all is entitled "Songs of Zion," the words on one page, the music on the other. It contains 200 pieces of music, for 75cts. The habitual singing of these hymns would bring blessings and happiness to any household.

"Blind Annie Lorimer" is a touching narration, and will mellow every heart which reads it; issued by the Presbyterian Board of Publication, 821 Chestnut St. Philadelphia, and by the Carter Brothers, 530 Broadway, New-York.

The American Tract Society, 28 Cornhill, Boston, and 13 Bible House, New York, have published "A Soldier of the Cumberland," with his portrait, being a memoir of Mead Holmes, jr., Sergeant of Company K. 21st Wisconsin Vol., by his Father, with an introduction by that eminent scholar and forcible writer, John S. Hart, L.L.D., a book full of facts and written in tears, deserving a wide circulation in the army; as also "How to be a Hero," by E. L. E. "John Freeman and his Family" is full of fun, as premonished by the dancing individuals in the title page.

"Diseases of the Throat and Lungs, their pathology, symptoms, &c.," by H. J. Phillips, M.D., of the University of St. Andrews and Member of the Royal College of London, &c. &c., 70 pages 8vo, sent post-paid for fifty cents. The book contains much information of practical value to intelligent readers; it very properly does not pretend to show invalides how to cure themselves, but rather informs them of the nature, causes, symptoms and general principles of cure, of the various ailments peculiar to the respiratory organs. Those who have not read much on those subjects will find a great deal that is instructive in this well got up publication.

"TOGETHER." Mr. Carlton will issue for the Holidays a new novel by the writer of "Olie" and "Nepenthe." Pure in its teachings, powerful in its delineations, the intelligent and the good will everywhere give it a hearty greeting. The loving and beautiful tribute which it gives to Professor Mitchel, should commend it to the perusal of every one of the tens of thousands who has been thrilled with the eloquence of the great astronomer and the noblest hero of his time.

THE NEXT YEAR. The subjects which will be treated of in the Journal of Health for 1865 will be found on another page under the heading of "200 Health Tracts," of which there will be about twenty in each number during the year; besides other reading adapted to the times and seasons. Single Nos. Fifteen Cents, post-paid. Subscription price One Dollar and a half a year. Address,

Hall's Journal of Health, No. 12 Union Square, New-York.

BETTER THAN TURKEYS! A New Englander writes for a quantity of the Soldier's Number of Hall's Journal of Health (Nov. 1864) saying "I hope the self-sacrificing soldiers will all have an abundant Thanksgiving dinner of Turkey and I intended to contribute, but after reading the Journal which wife and I praise and give thanks for the manner and matter of every number, wife says "Why not send the Journal, while others send the Turkies." "I will:" it will do them a *lasting* good, and may be the means of saving many lives."

SURGERY. Attention is called to Professor Daniels' advertisement.

Bound Vols of 1864 are sent post paid for $1.50 or will be exchanged for the loose numbers of 1864 for forty cents, missing Nos. for 10cts. Subscribers who have failed to receive any No. will be supplied without charge.

GREAT ACCOMODATION. Persons at a distance who want anything purchased in the city and forwarded promptly at a small percentage on its cost can address Henry B. Price, 455 Broadway, New-York, whose business energy and integrity is well known.

The January No. of Hall's Journal of Health will contain articles on Inconsiderate Things, Healthfulness of Fruits, Poisonous Bites, How to cure a Cold, Causes of disease, Burying alive, Care of the Eyes, Travelling hints, Music Healthful, Young old people, Dyspeptia, Drunkenness, Uses of Ice, Rules for Winter, Walking Erectly, Manly carriage, Sleeping posture, Winter Shoes, Cure for Corns, To grow beautiful, Measles, Consumption, Sab. Physiology, Best hair wash, Wearing flannel, health a duty.

☞ One of the Finest Country Seats on the east bank of the Hudson, of forty acres, for $30,000 00, in perfect order, including furniture. Apply to W. W. Hall, 12 Union Sq., N.Y.

